# Editorial: Sports medicine and physical rehabilitation, volume II

**DOI:** 10.3389/fvets.2023.1122309

**Published:** 2023-03-24

**Authors:** Michael H. Jaffe, David Levine, Denis J. Marcellin-Little

**Affiliations:** ^1^Department of Clinical Sciences, Mississippi State University, Starkville, MS, United States; ^2^Department of Physical Therapy, University of Tennessee at Chattanooga, Chattanooga, TN, United States; ^3^Department of Veterinary Surgical and Radiological Sciences, University of California, Davis, Davis, CA, United States

**Keywords:** veterinary sports medicine, veterinary rehabilitation, rehabilitation, sports medicine, orthopedics, canine rehabilitation, equine rehabilitation

Interest in Veterinary Sports Medicine and Physical Rehabilitation is continuing to grow. Articles within the 1st volume of the eBook focused on Veterinary Sports Medicine and Physical Rehabilitation published by Frontiers in Veterinary Science have been viewed more than 95,000 times. Continued research and clinical care in the field of veterinary rehabilitation has expanded our knowledge in several areas. As with all research, discovery prompts new questions and helps deepen the knowledge in the field to enhance the care for our veterinary patients. This Research Topic issue addresses several new questions that authors tackled through articles ranging from case reports, reviews, and original research. The 2nd volume of the eBook includes 18 new articles by 74 authors focused on a variety of topics. We are confident that readers will find these articles clinically useful as well as thought-provoking.

In the current eBook, several articles examine the physiologic impact of exercise on sporting and working dogs. Each article poses questions that will enlighten readers about the physiologic effects and the physical risks of training and performing their activities. Markley et al. approached training issues of dogs competing in agility through an internet survey. The article described factors contributing to injuries that occur during the course of training and competition, including training to jump before skeletal maturity. Their findings may serve to guide trainers in selecting appropriate activities for dogs of all ages. Similar methods have been used by Sundby et al. through an internet survey to further enhance our knowledge of demographic risk factors for injury in canine athletes, while Fry et al. investigated factors influencing the incidence of injuries to the iliopsoas muscle. Iliopsoas injury is often challenging to diagnose and manage. Their research gives us guidelines for the chronic impact that this injury causes and provides information that will guide clinicians managing that problem.

Several other articles in the current eBook focus on factors affecting working and sporting dogs. Pogue et al. investigated the effects of jump height on forelimb landing forces in Border Collies that compete in agility competitions. Jumping is known to increase the risk of carpi and forepaw injury. No difference was found when comparing kinematics and peak forces resisted by forelimbs during standard jumps and jumps with reduced height. This information opens the door for further investigation of the causes and effects of forelimb injuries during jumping. Essner et al. described training methods in Swedish sporting and working dogs. The article highlights the effects of physical exposure and management routines and provides insight about appropriate warm-up routines before activity. The article by Lenfest et al. examined the relationship of serum thyroid concentrations in sled dogs retired from their sport. Sled dogs often have a low baseline thyroid concentration. Once retired, these dogs continue to maintain a lower than standard reference range value to their thyroid concentration. This paper should help guide clinicians in their decision-making for these patients with respect to diagnosis and potential treatment for hypothyroidism.

Rehabilitation medicine practices often treat patients recovering from thoracolumbar intervertebral disc herniation. Amaral Marrero et al. investigated the effect of thoracolumbar intervertebral disc extrusion surgery on static body weight distribution during the recovery period following surgery. They also evaluated the impact of intervertebral disc disease on muscle atrophy by quantifying girth measurements. Dogs with intervertebral disc disease shifted weight forward early after surgery. While that cranial weight shift decreased over the 3 months that followed surgery, the cranial weight shift remained at the end of the study. In another 12-week-long study evaluating dogs with myelopathies, Sedlacek et al. evaluated the benefits of physical rehabilitation in dachshunds with mild or moderate myelopathy of the T3-L3 vertebral column segment. Most dogs did well and only one dog in nine had a recurrence of myelopathy within 2 years. In a pilot study, Lewis et al. described sensory-enhanced rehabilitation for patients with spinal cord injury. The study expanded our knowledge in that area of physical rehabilitation and offers opportunities to further investigate the effects of flooring and sensory stimulation on the recovery of neurologically-compromised dogs.

Osteoarthritis is a ubiquitous problem in dogs. Managing patients with osteoarthritis in practice remains very challenging. Physical rehabilitation and regenerative therapy are components of the multimodal management of canine osteoarthritis. The canine osteoarthritis staging tool (COAST) has been proposed to guide veterinarians and pet owners when diagnosing of osteoarthritis in its early stages. Mosley et al. proposed a consensus statement for Canadian veterinarians that is based on stages 1–4 of the COAST. The information will assist clinicians when they develop therapeutic plans for dogs with osteoarthritis. Kim et al. reported the result of an exploratory, double blinded, randomized, prospective clinical trial that compared the effects of allogeneic mesenchymal stem cell injection and to high-molecular-weight hyaluronic acid in dogs with osteoarthritis. Hyaluronic acid was more effective than stem cell injection in that study.

Muscle injuries are common injuries managed in physical rehabilitation and sports medicine. Their diagnosis can be challenging. This eBook includes a study in the horse that evaluated multifidus muscle function during exercise. Ursini et al. used electromyography to investigate the multifidus m. as a sentinel muscle which has been noted to atrophy due to chronic limb dysfunction. By measuring electromyographic changes in the multifidus muscle during a variety of therapeutic exercises, incorporating ground poles during exercise was effective in activating the multifidus muscle.

In a case report describing the management of infraspinatus and supraspinatus tendinopathy in two dogs, Owen documented tendon healing in dogs being managed using piezoelectric shockwave therapy. The paper adds to our knowledge regarding the use of shockwave therapy to manage tendon injuries in the dog shoulder. Weber et al. examined muscular activity in the forelimbs of retrievers carrying varying weights in their mouths while trotting. By evaluating dogs trotting across a pressure-sensitive walkway, they learned that the amount of pressure placed on the forelimbs increases when carrying heavier weights. The contraction time of the deltoideus muscle increased but contraction time in the biceps brachii muscle did not increase. This novel investigative approach to the bicipital tenopathy was informative and open the doors to future research.

Beyond these articles, several other articles provided original important information pertinent to physical rehabilitation: Frye et al. investigated strategies required to develop a treatment plan to provide physical rehabilitation to geriatric dogs, Rosen et al. prospectively evaluated complications in patients using orthoses or exoprostheses, Gundersen et al. reported a stifle function score and compared its association with ground reaction forces in dogs with cranial cruciate ligament rupture., and Bieber et al. measured ground reaction forces in dogs wearing protective footwear during training and exercise.

This second eBook volume on Sports Medicine and Physical Rehabilitation will be a valuable resource for rehabilitation and sports medicine clinicians. The Editors are extremely pleased with the strength and diversity of the 18 excellent articles included in this volume which will lay the groundwork for future studies and pose new questions in the field.

## Author contributions

All authors listed have made a substantial, direct, and intellectual contribution to the work and approved it for publication.

